# Current status, challenges and prospects of antifouling materials for oncology applications

**DOI:** 10.3389/fonc.2024.1391293

**Published:** 2024-05-08

**Authors:** Yingfeng Zhang, Congcong Sun

**Affiliations:** University-Town Hospital of Chongqing Medical University, Chongqing, China

**Keywords:** antifouling materials, cancers, targeted diagnostics and therapy, challenges, application status

## Abstract

Targeted therapy has become crucial to modern translational science, offering a remedy to conventional drug delivery challenges. Conventional drug delivery systems encountered challenges related to solubility, prolonged release, and inadequate drug penetration at the target region, such as a tumor. Several formulations, such as liposomes, polymers, and dendrimers, have been successful in advancing to clinical trials with the goal of improving the drug’s pharmacokinetics and biodistribution. Various stealth coatings, including hydrophilic polymers such as PEG, chitosan, and polyacrylamides, can form a protective layer over nanoparticles, preventing aggregation, opsonization, and immune system detection. As a result, they are classified under the Generally Recognized as Safe (GRAS) category. Serum, a biological sample, has a complex composition. Non-specific adsorption of chemicals onto an electrode can lead to fouling, impacting the sensitivity and accuracy of focused diagnostics and therapies. Various anti-fouling materials and procedures have been developed to minimize the impact of fouling on specific diagnoses and therapies, leading to significant advancements in recent decades. This study provides a detailed analysis of current methodologies using surface modifications that leverage the antifouling properties of polymers, peptides, proteins, and cell membranes for advanced targeted diagnostics and therapy in cancer treatment. In conclusion, we examine the significant obstacles encountered by present technologies and the possible avenues for future study and development.

## 1 Introduction

A tumor is a medical issue that poses a significant threat to human life. Tumors exhibit significant variability and genetic instability and can have a multifaceted etiology. Tumor treatment approaches encompass surgery, chemotherapy, radiation, targeted medication therapy, gene therapy, immunotherapy, and more (1). Differentiation-inducing drugs are commonly used to treat precancerous lesions and effectively prevent tumor formation. This therapy is only appropriate for patients in the early stages of malignant transformation. The process of preparing the differentiation-inducing chemical is challenging; thus, the therapy’s stability and safety require more confirmation. It is challenging to combat the intricate and fluctuating tumor environment with a single treatment; thus, the creation of integrated anti-tumor medicines is unavoidable. Tumor surgery has extended the lives of some patients and improved their quality of life to some degree, but it still has significant limitations.

Nanoparticles (NPs) are widely used in medicine and pharmacy due to their ability to effectively bypass biological defense systems and circulatory barriers. The applications encompass drug and gene delivery (2), growth and differentiation factor delivery in regenerative medicine (3), vaccination (4), fluorescent biological labelling (5), detection of proteins and pathogens, probing the DNA structure (6), separation and purification of biological molecules and cells (7), contrast agents in imaging, and phagokinetic studies (8).

Nanomaterials have diverse applications and can interact with biological systems due to their small size. They have the ability to bind with many functional units like diagnostic, targeted, and therapeutic molecules and can be directed to almost any physiological area (9). An example is a nanomaterial that is specifically engineered to extend the duration of blood circulation, avoid being taken up by macrophages, and ultimately reach the desired tissue (10). Upon entering a biological environment, a nanomaterial’s surface will be coated by layers of protein to create the protein corona (11). Protein corona formation is affected by factors such as the size, charge, surface modification of nanomaterials, exposure time, and protein components (12). The protein corona changes the physical and chemical properties of the nanomaterials, giving them a biological trait that differs from their original nature (13).

Biofouling is the unintentional and nonspecific attachment of biomolecules, cells, or microorganisms to material surfaces (14). Serum proteins in the blood play a key role in biosorption, leading to the phagocytosis and digestion of foreign substances by reticuloendothelial systems (RES) (15). Nanomedicines may experience a significant reduction in their circulation time in the body, leading to an inadequate therapeutic outcome (16). Protein adhesion occurs through electrostatic and hydrophobic interactions between charged segments and non-hydrophilic pockets in proteins (17). Several methods have been explored to enhance antifouling properties. The key principle in designing antifouling materials is to prevent electrostatic and hydrophobic interactions with biomolecules. This typically involves maintaining a neutral surface charge, high hydrophilicity, and having hydrogen bond acceptors but no hydrogen bond donors (18).

In this review, we introduce the most frequently used anti-fouling materials, describing their principles and giving examples. Moreover, an outlook on the future of anti-fouling targeted diagnostics and therapy for cancers is also presented.

## 2 Antifouling materials

Scientists prioritize modifying nanoparticles to mimic the body’s own cells in order to prevent them from being recognized as alien materials and eliminated by the immune system. Various methods have been and are now being implemented to transform nanoparticles into biocompatible substances. Efforts have been made to cover nanoparticles with non-reactive polymeric materials to prevent interaction with the host’s immune cells, creating a stealth effect that helps the nanoparticles avoid triggering the host’s immune response (19). Nanomaterial cloaking can use either natural or semisynthetic coverings. Natural polymers commonly used include dextran, polysialic acid, hyaluronic acid, chitosan, and heparin. Synthetic polymers, on the other hand, are man-made and include polyvinyl pyrrolidone, polyacrylamide, polyethylene glycol, and PEG-based copolymers like poloxamers, poloxamines, and polysorbates.

PEGylated nanoparticles are vulnerable to oxidative damage and reactive to transition metal ions in biological environments, leading to adverse consequences such as nonspecific protein binding and nanoparticle instability (20). Zwitterionic polymers offer advantageous features such as high hydrophilicity, strong nonfouling ability, biomimetic capabilities, and outstanding stealth characteristics as substitutes for PEGs in NP formulations (21). Several zwitterionic polymers have been created, such as poly (sulfobetaine ethacrylate), poly (2-methacryloyloxyethyl phosphorylcholine), poly (carboxybetaine methacrylate), and their related forms (22, 23).

### 2.1 PEG

Polyethylene glycol (PEG) is a non-toxic and biocompatible polymer. Polyethylene glycol (PEG) and its derivatives have been commonly used as anti-fouling materials since the 1970s (24). Poly (ethylene glycol) (PEG) modification is a commonly used method to decrease the nonspecific adsorption of proteins and cells. Molecules and nanoparticles modified with PEG exhibit extended blood circulation time and reduced nonspecific cellular uptake compared to unmodified materials, which is essential for specific targeting (25). PEG modification is a common technique used to minimize the binding of biofouling to surfaces (26). The feature, commonly referred to as the ‘stealth’ effect, is typically attributed to its low interfacial energy, the high degree of hydration of the hydrophilic polyether backbone, and the mobility and flexibility of the PEG chains (27).

Uniformly sized magnetite (Fe3O4) nanoparticles measuring 10, 20, and 31 nm were synthesized using the thermal breakdown of Fe (III) oleate or mandelate in a high-boiling point solvent (>320°C). The particles were coated with a PEG-containing bisphosphonate anchoring group to give them hydrophilic and antifouling characteristics. The PEGylated particles were analyzed using several physicochemical techniques such as dynamic light scattering, transmission electron microscopy, thermogravimetric analysis, Fourier transform infrared spectroscopy, and magnetization tests. Increasing the particle size from 10 to 31 nm resulted in a drop in the PEG coating quantity from 28.5 to 9 wt%. The PEG created a compact, brush-like layer on the particle’s surface, preventing particle aggregation in water and PBS (pH 7.4) and enhancing circulation time *in vivo*. Magnetic resonance relaxometry verified that the PEG-modified Fe3O4 nanoparticles exhibited strong relaxivity, which rose as the particle size grew. The particles caused noticeable contrast enhancement in the magnetic resonance imaging during *in vivo* investigations using a mouse model. Approximately 70% of the administered 20-nm magnetic nanoparticles remained in the bloodstream after four hours. However, their accumulation in the tumor was minimal, possibly because of the anti-fouling characteristics of PEG (28).

**Figure 1:**
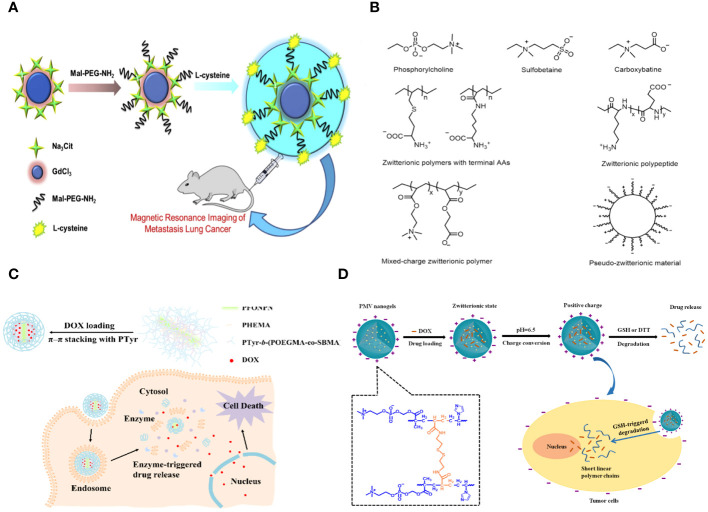
**(A)** Schematic representation of the synthesis of the Gd2O3- PEG-L-Cys-NPs. The Gd2O3- PEG-L-Cys-NPs *in vitro* application of MRI of metastasis lung cancer (29). **(B)** Models and structures of zwitterionic materials. **(A)** Chemical structures of the common zwitterionic groups. **(B)** Zwitterionic poly (amino acids) and polypeptide. **(C)** Mixed-charge zwitterionic polymers that have balanced cationic and anionic groups in different monomer units, and pseudo-zwitterionic materials with equimolar negative and positive charge binding to the same medium. **(C)** Schematic representation of the synthesis, drug loading, cellular uptake, and rapid enzyme-responsive drug release of the zwitterionic conjugated bottlebrush copolymers (30). **(D)** Illustration of the preparation, charge-conversion ability and biodegradable behavior of poly (2-methacryloyloxyethyl phosphorylcholine-s-s-vinylimidazole) (PMV) nanogels (31).

Yuan Sui and colleagues describe the creation and analysis of antifouling Gadolinium oxide (Gd2O3) nanoparticles (NPs) that have been altered with PEG to enhance biocompatibility for MR imaging. This article discusses the solvothermal breakdown of gadolinium (III) in the presence of Na3cit, observed through surface modification using PEG and L-Cys. The nanoparticles were verified using transmission electron microscopy (TEM), dynamic light scattering (DLS), and UV-visible spectroscopy. The morphological analysis indicates that the perfect Gd2O3-PEG-Cys-NPs have a consistent distance of 7.9 ± 0.4 nm, with minimal variation in size. This suggests that the surface modification does not significantly change the core size of the Gd2O3-NPs compared to the pristine sodium citrate-stabilized Gd2O3-NPs. The Gd2O3-PEG-L-Cys-NPs exhibit great stability at room temperature, are water dispersible, and demonstrate reduced cytotoxicity at high concentrations. The T1-weighted MR images clearly showed that the PEG-coated Gd2O3-PEG and Gd2O3-PEG-Cys-NPs, with or without Cys, had the potential to be used for T1-weighted MR imaging. The nanoparticles show no toxicity towards human blood, indicating their biocompatibility for medical uses in humans. The Gd2O3-PEG-Cys-NPs exhibit excellent r1 values, good cytocompatibility, target particular cancer cells, and enable dual-mode MR imaging of lung metastasis cancer models *in vitro* (**Figure 1A**) (29).

Reportedly, PEGylations lose their protein repellent ability at temperatures over 35°C (32). PEG is vulnerable to oxidation when exposed to oxygen or transition metal ions, causing damage to its non-ionic structure and leading to loss of function in many biochemically significant solutions (33). Moreover, PEG has been shown to be challenging to naturally metabolize, and multiple injections of PEGylated formulations can decrease the bioactivity of enclosed biomolecules because of antibodies generated by the immune system (34). The gene entrapment efficiency of PEGylated cationic liposomes would decrease because of the reduction in positive charges (35). Therefore, it is essential to identify alternative materials that can withstand biosorption during blood circulation, remain stable in various environments, be metabolized through biodegradation, and have minimal immunogenicity apart from PEG.

### 2.2 Zwitterions

Zwitterions, which have both positive and negative charged groups, create a neutral charge. They are thought to be better at keeping things from sticking to nanomaterial surfaces for cancer diagnosis than PEG or OEG. Nanomaterials that have been changed zwitterionically have a surface with an equal number of negative and positive groups. This lets a lot of water molecules stick to the surface as a hydration layer through hydrogen bonding. This results in a highly hydrophilic surface that protects the materials from nonspecific protein pollution (36).

Polyzwitterions can be fabricated into various structural forms such as brushes (37), films (38), hydrogels (39), particles (40), membranes (41), and coatings (42), each serving different functions like antifouling (43), stimuli responsiveness (44), lubrication (45), self-healing (46), antibacterial properties (43), and biosensing capabilities (47). Because of their high tolerance to extremely salty conditions, these materials have the potential to be used in various applications such as ionomers (48), fibers (49), rheology modifiers (50), drug/gene delivery vehicles (51), analogues of biological structures (52), and anti-fouling materials (53). Zwitterionic materials not only have excellent antifouling properties but also improve biocompatibility, decrease immunological response, facilitate cellular uptake of chemical drugs and genes, and extend circulation time (54). Zwitterionic alterations can offer unique capabilities as drug transporters, including responsiveness to stimuli and targeting of tumors (55).

Zwitterions can be categorized as betaine-like zwitterions or mixed-charge zwitterionic materials based on the location of the cationic and anionic groups on the same unit (**Figure 1B**). Zwitterionic polymers predominantly contain quaternary ammonium as cations, forming phosphorylcholine (PC), sulfobetaine (SB), and carboxybetaine (CB) with phosphonates (PO3-), sulfonates (SO3-), and carboxylates (COO-) accordingly. Aside from zwitterionic polymers with charged segments on the same side chains, there are mixed-charge materials with equal positive and negative charged components in separate monomer units or attached to the same medium (such as mesoporous silica nanoparticles) to ensure overall electrical neutrality. These ‘spurious’ zwitterionic materials possess comparable antifouling capabilities due to their similar architectures. Furthermore, researchers have successfully incorporated amino acids or peptides to create zwitterionic carriers, which are a type of natural zwitterions, demonstrating excellent resistance to nonspecific absorption and possessing distinctive features.

Fangjun Liu et al. created enzyme-responsive theranostic zwitterionic bottlebrush copolymers with brush-on-brush architecture. The copolymers include a fluorescent PFONPN backbone that interacts with DOX through fluorescence resonance energy transfer, primary PHEMA brushes, and secondary graft brushes with enzyme-degradable PTyr side chains and zwitterionic P(OEGMA-co-SBMA) side chains. They achieved this by combining Suzuki coupling, NCA ROP, and ATRP techniques. The brush-on-brush copolymer created has a particular response to the tumor microenvironment. It can create individual micelles in water with a high drug capacity due to the extremely hydrophilic zwitterionic brushes. This copolymer serves as an innovative nanoplatform for cancer treatment and diagnosis (**Figure 1C**) (30).

Biodegradable nanogels made of poly (2-methacryloyloxyethyl phosphorylcholine-s-vinylimidazole) (PMV) were created by a single-step reflux precipitation polymerization process, resulting in homogeneous spherical shapes. The method was both clean and efficient. The PMV nanogels maintained a zwitterionic state at pH 7.4 and transitioned quickly to a positively charged state at pH 6.5 in the tumor extracellular environment. The charge-conversion capacity of PMV nanogels was demonstrated by proton nuclear magnetic resonance spectra and an acid-base titration experiment, which showed that the imidazole ring becomes protonated in an acidic environment. The protein stability experiment demonstrated that PMV nanogels showed resistance to protein adsorption at pH 7.4 for up to 7 days but readily adsorbed protein at pH 6.5. Also, PMV nanogels had a property called “reductibility,” which meant they could break down into shorter linear polymer chains when they were exposed to reducing agents. Hence, the doxorubicin (DOX) release was precisely regulated, with minimal leakage under normal circumstances (7.8% in 48 hours) and rapid release in 10 mM glutathione at pH 7.4 (78.9% in 48 hours). The PMV nanogels demonstrated increased cellular absorption by tumor cells at pH 6.5 compared to pH 7.4, leading to a strong cytotoxic effect of DOX-loaded PMV nanogels against tumor cells, as observed using confocal laser scanning microscopy and flow cytometry (**Figure 1D**) (31).

### 2.3 Proteins and peptides

Albumin is often used as a blocking agent to keep background signals from messing up different types of experiments, such as immunocytochemistry and Western blot. The antifouling capability is due to the distinctive composition of amino acids in the albumin structure, resulting in a well-balanced charge distribution (56).

Functionalized anti-fouling peptides are popular as an anti-fouling material due to their natural biocompatibility and are commonly used in an electrochemical assay for tumor markers. Peptides have significant hydration properties due to their polar functional groups and zwitterionic charges, which contribute to their anti-fouling effect. Peptides that are hydrophilic and amphiphilic but lack charge exhibit anti-fouling qualities. An example is the anti-fouling portion of peptides containing the EK motif, which was engineered to be electrically neutral (57). Neutral or hydrophilic anti-fouling peptides with certain functional groups can be created. Peptides provide numerous benefits, making them an excellent choice for biodegradable anti-fouling compounds (58).

Peptides can form precise nanostructures that are beneficial for targeting in biological systems, although they exhibit low bioavailability, possible immunogenicity, and inadequate metabolic stability. Peptidomimetic self-assembled nanoparticles can contain biological recognition patterns while also offering certain technical characteristics. Inorganic nanoparticles, covered with self-assembled macromolecules to enhance stability and prevent fouling and linked with ligands unique to a target, are improving imaging resolution from anatomical to molecular levels. Nanoparticles conjugated with ligands are appealing for delivering drugs selectively to cells in tumors due to their high transport capacity and cell selectivity based on the ligand. Peptidomimetic nanoparticles can enhance binding to surface receptors on cancer cells, leading to increased uptake and decreased drug resistance. Self-assembled nanoparticles linked with peptidomimetic antigens are optimal for prolonged display of vaccination antigens to dendritic cells, leading to activation of the T-cell-mediated adaptive immune response. Self-assembled nanoparticles are a feasible substitute for encapsulation in providing prolonged release of proteins in tissue engineering. Cell-penetrating peptides attached to nanoparticles are used as vectors for intracellular delivery of genes and as agents for transferring plasmids (59).

**Figure 2:**
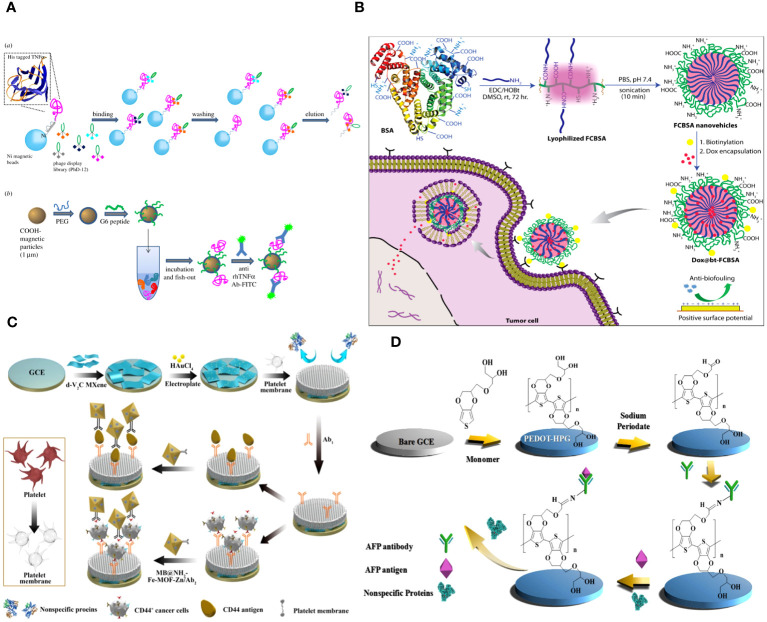
**(A)** Graphical scheme for the design and development of MP-based bioassay for fishing-out of soluble biomarkers. (a) Experimental scheme for selection of phage-displayed peptides with high affinity and specificity for rhTNFa performed on magnetic nickel-coated beads. (b) Graphical representation of integrated system for detection or fishing-out of any soluble biomarker in complex biological medium. This MP-based bioassay consists of selective capturing of target protein due to the presence of a specific binding peptide (previously selected by modified phage display procedure). The binding event is then detected by immunofluorescence measurements (60). **(B)** Schematic representation of the synthesis of fatty-amine-conjugated cationic BSA nanovehicles formulation, its surface modification with biotin, the capacity for antibiofouling, and successful encapsulation and delivery of anticancer drug Dox to biotin-receptor-positive cancer cells (61). **(C)** The fabrication process of the electrochemical immunosensor with anti-fouling capability for detection of CD44 (62). **(D)** Schematic illustration of the fabrication process of the AFP biosensor with PEDOT-HPG (63).

Angela Maria Cusano and colleagues discuss a universal method for directly detecting a particular tumor biomarker in serum. Detection is enabled via a protein-binding peptide chosen through an enhanced phage display method and then attached to modified microparticles (MPs). Protein biomarkers provide abundant data for non-invasive diagnostic and prognostic studies. MP-based assays are increasingly used for handling soluble biomarkers; however, their application in serum is hindered by the intricate biomolecular surroundings. Their method surpasses the existing constraints by creating a selective MP with an anti-fouling layer that effectively captures the target protein while remaining unaffected by other substances in the background. Their technique effectively isolates human tumor necrosis factor alpha from serum with a high level of selectivity (**Figure 2A**) (60).

Abhishek Saha and colleagues describe a straightforward method for creating fatty-amine-conjugated cationic BSA (FCBSA) nanoparticles by attaching laurylamines to the BSA protein. When glutamic acid or aspartic acid residues partially neutralize by forming an amide bond with laurylamines, cationic nanoparticles are produced under physiological conditions. These nanoparticles have an isoelectric point of 7.7 and a zeta potential of +7 mV at pH 7.2. The NPs demonstrate strong resistance to heat, pH, and proteolytic enzyme stressors. The NPs showed outstanding biocompatibility with normal and cancer cell types. The protein NPs effectively trap the hydrophobic anticancer medication doxorubicin (Dox) and exhibit controlled release characteristics (about 40% release after 3 days), stability in human blood serum, resistance to fouling, and a stronger attraction to anionic membranes. The biotin-labelled cationic FCBSA (bt-FCBSA) exhibited nearly identical biophysical characteristics as FCBSA. Additionally, cellular investigations demonstrated that bt-FCBSA effectively transports Dox to biotin receptor-positive HeLa cells, resulting in substantial cell mortality. An *in vivo* study showed that Dox-encapsulated bt-FCBSA significantly inhibited tumor growth in female Swiss albino mice with Ehrlich ascites carcinoma cells (**Figure 2B**) (61).

### 2.4 Cell membrane

The cell membrane, consisting of phospholipid bilayers and proteins, acts as a semipermeable barrier to prevent large biomolecules from entering the cell (64). Many biomimetic platforms were created by coating materials with cell membranes to replicate this characteristic. These materials, camouflaged with cell membrane, possess biocompatibility, low immunogenicity, extended blood circulation time, and antifouling properties due to inheriting the natural qualities of the cell membrane (65). Cell membrane-coated materials have demonstrated potential uses in medication delivery, imaging, and cancer diagnostics.

The cell membrane demonstrates excellent biocompatibility. The hydrophilic phospholipid head was exposed to the sample because of the topology of the phospholipid bilayer. Meanwhile, the head of the phospholipid is composed of a negatively charged phosphate group and a positively charged quaternary ammonium group, resulting in overall surface electroneutrality (36, 66). The antibacterial fouling ability of the PM/AuNPs/d-V2C-modified electrode relies on the steric hindrance of glycoproteins, the presence of zwitterionic headgroups in phospholipid bilayers, and the high hydrophilicity of the interface (**Figure 2C**) (62).

Red blood cells are the most prevalent type of blood cell in the circulatory system. RBC membrane-based materials have significant potential in clinical applications such as drug delivery, immunological evasion, tumor imaging, and cancer diagnostics due to their biocompatibility, biodegradability, long circulation half-life, and anti-nonspecific adsorption ability (67).

White blood cells (WBCs) play a crucial role in various serious illnesses, including infections, cancer, and inflammatory disorders. They also aid in immunological functions and build up in different diseased regions. Given that isogenous white blood cells (WBCs) do not participate in the cluster reaction within the same environment, the utilization of nanoparticles based on the membrane camouflage of WBCs, such as neutrophils, macrophages, and T cells, can effectively mitigate nonspecific leukocyte binding. This is achieved by coating the membrane of WBCs with nanoparticles. Consequently, the background of white blood cells (WBCs) might be reduced, leading to an enhancement in the purity of CTC capture. Consequently, multiple platforms containing membrane-coated nanoparticles for white blood cells (WBCs) have been created to isolate cancer stem cells (CTCs).

In addition to red blood cells (RBCs), many types of cells have been considered based on the specific therapeutic purposes required. These include RBCs, white blood cells (WBCs), platelets, stem cells, cancer cells, and other non-traditional sources. The material cores considered in this study include biodegradable polyester nanoparticles (PLA, PLGA, and PCL), mesoporous silica nanoparticles, magnetite nanoparticles, gelatin, and ultra-caprolactone nanoparticles (UCNPs). The encapsulation of cancer cells within PLGA nanoparticle cores has been employed for the purpose of targeting melanoma (68). Additionally, stem cell-coated PLGA nanoparticles have been utilized for the effective targeting of orthotropic breast cancer (69). Furthermore, macrophage-coated mesoporous silica nanoparticles have been employed as a biomimetic platform (70). Neutrophil-coated nanoparticles (NPs) have been developed as a means of delivering chemotherapeutic medicines for the treatment of recurrent glioblastoma. Additionally, these nanoparticles have shown potential for suppressing inflammation of the synovial membrane and mitigating joint damage in individuals with inflammatory arthritis (71). The toxins secreted from staphylococcus, specifically alpha-hemolysin, were absorbed by nanoparticles coated with RBC membranes (72). In addition to the aforementioned, various other NP cores can be utilized for extensive therapeutic applications. In their study, Yang et al. (2019) developed a cancer cell membrane shell and a core composed of silica nanoparticles. These components were designed to encapsulate a photodynamic agent, chlorin e6, with the aim of establishing an efficient photodynamic therapy (73). Gold nanoparticles coated with lipids have been utilized in many applications, such as drug transport, diagnostics, and sensing (74).

Hu et al. did a study on how to use PD-1 receptor-presenting membrane-coated paclitaxel dimers nanoparticles (PD-1@PTX2 NPs) to make treatment work better. Shrouded on the PD-1 cell membrane, the PTX dimer demonstrated efficient cellular absorption and enhanced cytotoxicity against cancer cells. PD-1@PTX2 nanoparticles have the ability to specifically attach to PD-L1 ligands that are present on breast cancer cells. The nanoparticles demonstrate a notable rate of tumor growth reduction, namely 71.3%, in mice that hold 4T1 xenografts. Additionally, these nanoparticles greatly extend the survival period in animal models of breast cancer. Furthermore, our nanoparticles made it possible for 3.2 times more CD8+ T cells to enter tumors and a 73.7% drop in the number of regulatory T cells (Tregs) to be present, which strengthened the immune response against tumors. The results underscore the potential application of PTX nanoparticles on immune checkpoint receptors to enhance the efficacy of chemoimmunotherapy. This offers an additional method for enhancing cancer treatment (75).

### 2.5 Other anti-fouling materials

Hydrogels are interconnected structures made of hydrophilic polymers that include many hydrogen bonds. Hydrogels have a high specific surface area and a distinctive three-dimensional network structure, allowing them to hold a variety of modified materials for electrode modification. It is possible for hydrogels to hold many molecules with different functions inside them, giving them high conductivity, strong electrochemical signals, and great catalytic performance. Simultaneously, the microenvironment within hydrogels can enhance the stability and biological function of biomolecules. Moreover, the high permeability of hydrogels can expedite the movement of tiny molecules and ions, as well as the quick transfer of electrons. Hydrogels have unique features that make them highly promising for constructing electrochemical immunosensing surfaces (76).

A new redox hydrogel called PANI-PThi gel was created for very sensitive and protein-repellent amperometric immunosensing of carcinoma antigen-125 (CA12–5). The PANI-PThi gel-modified electrode underwent an anti-fouling performance test by being submerged in a PBS solution containing various serum concentrations for 12 hours. The current fluctuation range did not exceed 4 µA, demonstrating the PANI-PThi gel’s ability to notably decrease the non-specific adsorption of proteins in human serum. The SWV curves showed that the suggested hydrogel has a substantial and consistent signal, maintaining 94.6% even after a month. The response of PANI-PThi gels is enhanced by the presence of H2O2, leading to an extended linear range for the immunosensor. The biosensors’ results aligned closely with ELISA when detecting clinical serum samples (77).

The following studies examined how branching impacts the blood circulation and tumor targeting of polymer nano vehicles in living organisms. Star-branched copolymers of poly (lactic acid) and poly (2-methacryloyloxyethyl phosphorylcholine) (PLA-PMPC) were synthesized with umbrella-type AB3, (AB3)2, and (AB3)3 architecture by branching at the PLA core for the intended application. Micelles formed from these copolymers were used to study the impact of core branching on blood circulation and tumor targeting. Branching altered the polymeric self-assembly in solution, leading to modifications in the size and surface anti-fouling properties of the polymeric micelles, as indicated by the results. Star-branched copolymer micelles with increased branching degree improved the persistence of their payload in blood, extending the half-time from 7.1 and 8.6 hours to 13.8 hours and resulting in 1.72 times more content at the tumor site. The research indicates that increasing the branching degree of amphiphilic copolymers could be a beneficial approach for creating carriers with improved circulation and targeting abilities in living organisms (78).

Li et al. developed a new antifouling platform named Fe3O4@SiO2@PTMAO@Aptamer by attaching polymeric trimethylamine N-oxide (PTMAO) onto Fe3O4@SiO2 nanoparticles and then linking it with two aptamers for capturing circulating tumor cells (CTCs) (79).

Hyperbranched polyglycerol (HPG) exhibits high hydrophilicity due to its dense, spherical structure containing several hydroxyl groups. PEDOT was polymerized with HPG using electrochemical polymerization to enhance conductivity. The hydration layers generated on the surface of PEDOT-HPG are a result of the many hydroxyl groups present, which prevent protein adsorption and discourage non-specific cell adhesion. The study demonstrated that the biosensor exhibited strong resistance to fouling and high sensitivity, with a detection limit of 0.035 pg mL^-1^ (**Figure 2D**) (63).

## 3 Application of antifouling materials in oncology

### 3.1 Improvement of tumor diagnosis

Cancer is still regarded as one of the most lethal diseases. Prompt diagnosis and therapy are now the preferred methods to enhance the survival rate of cancer patients. Accurate medical diagnosis of tumor characteristics, such as its location, boundaries, and spread to other areas, is crucial for determining appropriate treatment. Several diagnostic methods rely on contrast agents to improve imaging efficiency, resolution, and precision, including fluorescence imaging, magnetic resonance imaging (MRI), computed tomography (CT), and radioisotope-based nuclear imaging techniques. Nanomaterials have gained attention due to the limitations of traditional contrast agents with low molecular weights, such as high concentration-induced renal toxicity, low imaging efficiency, rapid metabolic pathway-induced short imaging time window, and lack of specificity. Examples include Omnipaque for CT imaging and Magnevist for MR imaging. This is mostly due to their inherent features that can be utilized for specific surface modifications to produce adjustable pharmacokinetics and as bases to incorporate imaging agents or pharmaceuticals for diagnostic and therapeutic purposes (80).

Nanomaterials are commonly used as platforms to carry imaging agents for cancer detection. Typically, most nanomaterials tend to accumulate in organs related to the reticuloendothelial system (RES), like the liver, spleen, and lungs, following systemic injection. While the method of modifying nanomaterials using polyethylene glycol (PEG) has somewhat alleviated the issue, obstacles persist for additional therapeutic uses. Nanomaterials modified with zwitterionic surfaces have demonstrated superior antifouling properties compared to those modified with PEGylation.

#### 3.1.1 Antifouling materials in tumor imaging

Molecular imaging technologies like optical (81) computed tomography (82), magnetic resonance (MR) (83), positron emission tomography (PET) (84), and single-photon emission computed tomography (SPECT) (85) are used for disease diagnosis because of their inherent benefits (86). Magnetic resonance imaging (MRI) is widely used in clinical diagnosis for its exceptional resolution and tomographic abilities (87). Nanomaterial-based contrast agents are commonly used in MR imaging applications to enhance imaging sensitivity and reliability.

Over the last ten years, nanotechnology has rapidly advanced in creating and producing several types of nanoparticles for magnetic resonance imaging purposes. Gd (III)-loaded nanocarriers, such as dendrimers (88), liposomes (89), micelles (90), and inorganic NPs (91), provide significantly improved image contrast compared to tiny-molecule Gd (III) complexes. Furthermore, due to the extended blood circulation time and easy surface modification of NPs, NP systems can achieve increased imaging duration, desirable compatibility with living organisms, and enhanced imaging accuracy. Carbon nanotubes (CNTs) (92) are considered highly appealing because of their exceptional characteristics, including ultrahigh surface area, ultralight weight, outstanding chemical and thermal stability (93), and extended circulation duration, which make them stand out among other nanomaterials (94). Ultra-short single-walled carbon nanotubes (SWNTs) have been utilized as a high-performance T2-weighted magnetic resonance imaging (MRI) contrast agent in MR imaging applications. This is attributed to the paramagnetic properties of the SWNTs and the presence of iron catalyst nanoparticles.

**Figure 3:**
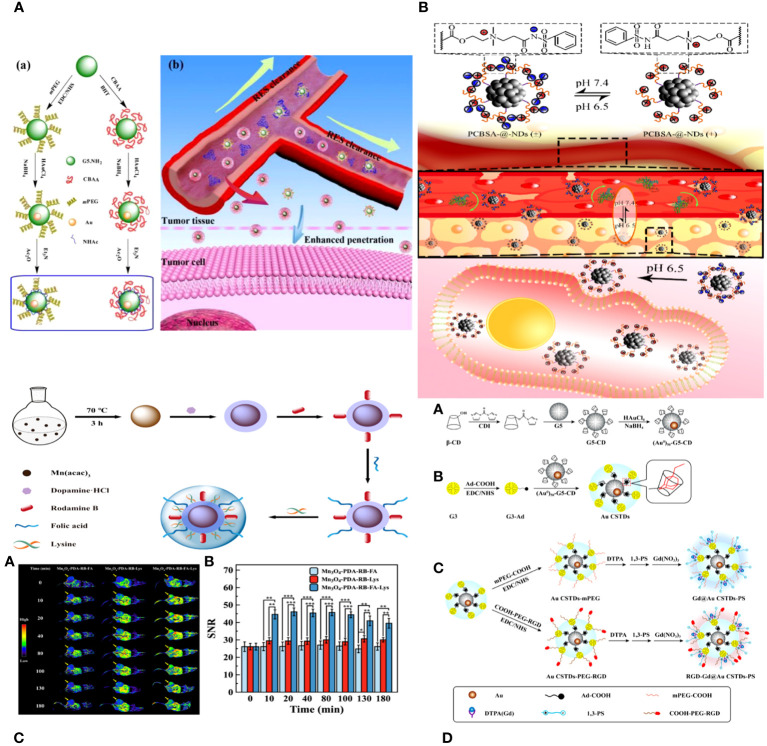
**(A)** Schematic illustration of the synthesis of CBAA- or PEG-modified Au DENPs (a) and the good antifouling property of CBAA-modified Au DENPs in blood vessels for imaging applications (b) (95). **(B)** The surface charge-conversional performance of PCBSA-@-NDs and tumor cell uptake under tumor pHe (96). **(C)** Schematic presentation of the preparation of Mn3O4−PDA−RB−FA−Lys NPs (97). **(D)** Schematic illustration of the synthesis of RGD-Gd@Au CSTDs-PS (98).

Zhijuan Xiong and colleagues describe the creation and analysis of antifouling zwitterion carboxybetaine acrylamide (CBAA)-modified dendrimer-entrapped gold nanoparticles (Au DENPs) for improved CT imaging purposes. The CBAA-modified nanodevice demonstrates superior protein resistance, reduced macrophage uptake and liver accumulation, and prolonged blood half-life compared to the PEGylated material, resulting in improved CT imaging of the blood pool, lymph nodes, and tumors (**Figure 3A**) (95).

Biyu ZHOU et al. developed a zwitterionic polymer coating on nano diamonds (ND) with pH-responsive properties for improved imaging of tumor cells using commercial NDs. The process began by grafting poly (carboxybetaine methacrylate) onto the virgin NDs using surface-initiated reversible addition-fragmentation chain transfer (RAFT) polymerization. PCBMA-@-NDs were replaced with benzene sulfonamide (PCBSA-@-NDs) using one-step carbodiimide chemistry to make them pH-responsive and enhance their interaction with tumor cells. The surface modification of the polymer was analyzed using FTIR, 1H NMR, and TGA. PCBMA-@-NDs and PCBSA-@-NDs exhibited improved dispersibility, increased fluorescence intensity, and superior antifouling properties compared to pristine NDs. Furthermore, PCBSA-@-NDs may reversibly convert its zwitterionic surface (at pH 7.4) to a positive charge (at pH 6.5) by protonating or deprotonating acylsulfonamide. PCBSA-@-NDs exhibited superior cell affinity and imaging performance compared to zwitterionic NDs in a slightly acidic tumor environment, as confirmed by fluorescence microscopy and flow cytometry (**Figure 3B**) (96).

Peng Wang and colleagues introduce the development of antifouling zwitterion-functionalized manganese oxide (Mn3O4) nanoparticles (NPs) that are coated with folic acid (FA) for precise imaging of tumors using magnetic resonance (MR) technology. Diethylene glycol-stabilized Mn3O4 nanoparticles were synthesized using a solvothermal method. They were then coated with polydopamine, labelled with rhodamine B for fluorescence, conjugated with folic acid through amide bond formation, and lastly coated with L-lysine zwitterions. The Mn3O4 NPs created in this manner exhibit superb water dispersibility and colloidal stability, effective protein resistance, and favorable cytocompatibility. Because of the PDA and Lys changes, the multifunctional Mn3O4 NPs have an extremely high r1 relaxivity of 89.30 mM^−1^ s^−1^. They also facilitate targeted tumor MR imaging by including FA ligands. The zwitterion-functionalized Mn3O4 nanoparticles can be used as a high-quality contrast agent for targeted magnetic resonance imaging of many biological systems (**Figure 3C**) (97).

Combining various imaging technologies is frequently utilized in clinical settings to enhance the accuracy of cancer diagnosis by overcoming the limitations of individual imaging modalities. Computed tomography (CT) is a commonly used imaging tool that provides detailed 3-dimensional images with high spatial and density resolution for anatomical structure and functional information (99). On the other hand, magnetic resonance (MR) imaging offers high sensitivity and excellent resolution for soft tissue (100). Thus, creating new CT/MR dual-mode imaging contrast agents could combine the benefits of both imaging techniques and enhance the precision and sensitivity of cancer detection (101).

Renna Liu and colleagues developed multifunctional core-shell tecto-dendrimers (CSTDs) containing gold nanoparticles (Au NPs) for dual-mode imaging of tumors using computed tomography (CT) and magnetic resonance (MR). β-cyclodextrin (CD)-modified generation 5 poly(amidoamine) (PAMAM) dendrimers were synthesized and encapsulated with Au NPs at the core in this study. Third-generation PAMAM dendrimers that were changed with adamantine worked as a protective layer to make Au CSTDs, which are threaded structures made of cyclodextrin and gold nanoparticles. This was done by adamantine and cyclodextrin interacting with each other on a supramolecular level. The Au CSTDs was replaced with RGD peptides that had PEG between them, along with a Gd chelator and 1,3-propane sultone. Gd (III) ions were then chelated. The synthesized Au CSTDs are multifunctional nanoparticles with a mean size of 11.61 nm. They are very stable as colloids, block X-rays well, have a high r1 relaxivity (9.414 mM^−1^s^−1^), are good at preventing fouling, and are compatible with cells. The multifunctional Au CSTDs makes it possible to use targeted CT/MR imaging of a breast cancer model in living organisms. This is possible because RGD helps target αvβ3 integrin-overexpressing cancer cells. They can be removed from the body through a metabolization pathway with an excellent biosafety profile. The advanced Closed System Transfer Devices (CSTDs) can be used as a precise imaging tool in both CT and MR modes to find different types of cancer that have high levels of the αvβ3 integrin (**Figure 3D**) (98).

#### 3.1.2 Application of antifouling materials in biosensors

Biosensors have been extensively utilized in several fields such as biotechnology, food inspection, medical diagnostics, and environmental monitoring since their inception by Lyons and Clark in the 1960s (102). Electrochemical biosensors are a very sensitive detection technology that offers the benefits of easy operation and cost-effectiveness (103). Currently, the extended implementation of electrochemical biosensors encounters two significant obstacles. One must determine how to implement it in clinical settings when faced with resistance to interference. The second issue is how to sustain high detection efficiency following the application of antifouling compounds. Utilizing electrochemical biosensors in clinical practice is currently a significant challenge due to the potential issue of nonspecific protein adsorption in complex biological samples like human whole blood. This can lead to decreased sensitivity and specificity in detection, resulting in false positive responses (104). Many approaches aimed at reducing background interference and nonspecific interactions have experienced significant progress (105). A sample dilution was used to reduce the background signals. Although effective in reducing the presence of non-target molecules, this method may not always be practical (106). Surface blocking has had significant success in targeting therapeutic sites in human physiological fluids by utilizing adsorbed and non-reactive substances like Tween 20, BSA (bovine serum albumin), and commercially available combinations (66). Polymeric separation membranes have made significant progress in minimizing nonspecific adsorption on gold surfaces, utilizing poly (ethylene glycol) (PEG) polymer (107), zwitterionic polymer (108), and peptide (109). Phosphocholine has emerged as a promising antifouling membrane material in recent years. Phosphocholine can imitate eukaryotic membrane properties (110), making it very resistant to protein or cell non-specific adsorption. This is achieved by creating a hydration layer through strong water binding (111). Phosphocholine-integrated layers are short, which increases the likelihood of fast electron transfer.

**Figure 4:**
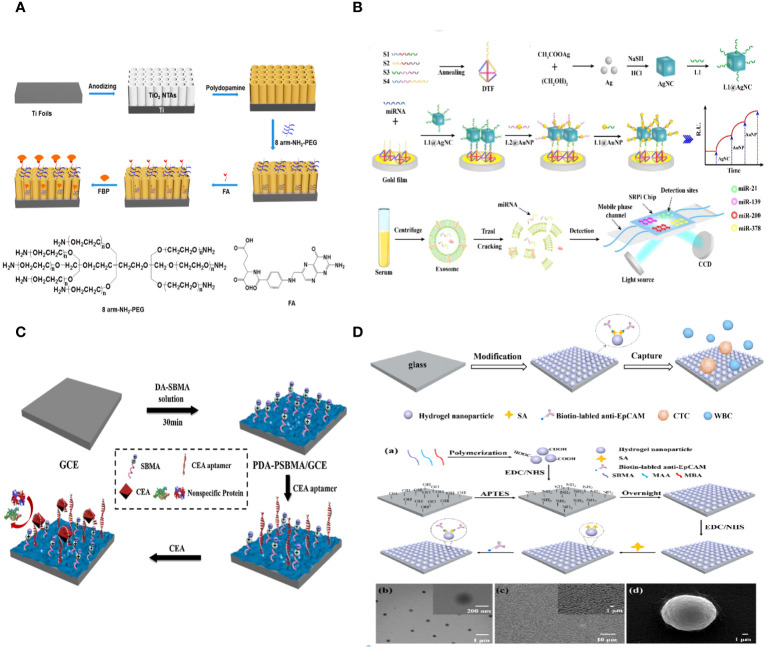
**(A)** Schematic Representation of the Fabrication of a PEC Biosensor for FBP Detection (112). **(B)** Schematic illustration of the multiplex exosomal miRNAs detection using SPRi biosensor (113). **(C)** Schematic illustration of the fabrication process of the PDA-PSBMA based sensing platform (114). **(D)** Schematic illustration for the capture of circulating tumor cells (CTCs) using the hydrogel nanoparticle substrate. Preparation and characterization of hydrogel nanoparticle substrate (115).

Serum-soluble folate binding protein (FBP) is a crucial tumor marker, and there is a strong demand for the creation of a straightforward biosensing technique. Bobo Fan et al. developed a photoelectrochemical (PEC) biosensor to detect FBP by creating an antifouling surface and utilizing unique ligand-protein recognition. The PEC sensing platform was created by covering TiO2 nanotube arrays (NTAs) with biomimetic polydopamine (PDA). The macroporous structures led to a substantial boost in PEC. Conjugating amino-group-terminated 8-arm polyethylene glycol (PEG) resulted in outstanding antifouling performance. Folic acid (FA) inclusion maintains the antifouling function and demonstrates recognition capabilities for FBP. The artificial photoelectrochemical biosensor demonstrates excellent analytical capabilities (**Figure 4A**) (112).

Exosomal miRNAs have the potential to serve as tumor biomarkers for the early detection of non-small cell lung cancer (NSCLC). A biosensor utilizing surface plasmon resonance imaging (SPRi) was created to identify several non-small cell lung cancers (NSCLC)-related exosomal miRNAs in a clinical sample. This biosensor combines an Au-on-Ag heterostructure with a DNA tetrahedral framework (DTF). Exosomal miRNAs are trapped by different DNA-templated fluorescent probes fixed on the gold array chip. The ssDNA-functionalized silver nanocube hybridizes with collected exosomal miRNAs. Subsequently, ssDNA-coated Au nanoparticles assemble on the AgNC surface, creating Au-on-Ag heterostructures that act as crucial markers for increased SPR response. The SPRi-based biosensor, utilizing DNA-programmed Au-on-Ag heterostructure and DTF, demonstrates a broad detection range of 2 fM to 20 nM, an extremely low limit of detection of 1.68 fM, increased capture efficiency, and enhanced antifouling capacity (**Figure 4B**) (113).

A new method was created to build highly sensitive and resistant biosensors that can detect the tumor marker CEA in complex biological samples. This method involves a one-step copolymerization of polydopamine (PDA) and poly (sulfobetaine methacrylate) (PSBMA). When copolymerized PDA and PSBMA are present, CEA aptamers with thiol groups can be connected to the PDA through the Michael addition reaction. The zwitterionic polymer PSBMA helps maintain the antifouling properties of the sensing interface. The electrochemical biosensor demonstrated effective efficacy in detecting CEA, with a linear range of 0.01–10 pg/mL and a low limit of detection (LOD) of 3.3 fg/mL. The presence of PSBMA in the sensing interface allowed the electrochemical biosensor to detect CEA in complex biological samples with a good antifouling effect, demonstrating the potential of biosensors using copolymerized PDA and PSBMA for analyzing human serum samples (**Figure 4C**) (114).

#### 3.1.3 Application of antifouling materials in CTCs

Circulating tumor cells (CTCs) are a small number of cancer cells that move from the primary or metastatic tumor site into the bloodstream and play a crucial role in cancer metastasis, which is the primary cause of cancer-related mortality (116). CTC detection, a non-invasive approach, could allow for early cancer diagnosis, prognosis, real-time treatment monitoring, and the identification of new treatment targets. Circulating tumor cells (CTCs) are cells that are released from solid tumor tissue into the bloodstream and are being studied as a diagnostic for early cancer diagnosis and prognosis. Various systems have been created to isolate circulating tumor cells due to their potential importance in clinical settings. Yet, effectively isolating CTCs poses considerable hurdles, particularly in attaining the required sensitivity and specificity because of their great scarcity and severe biofouling in blood, which includes numerous background cells and diverse proteins. Recent advancements in CTC detection technology have led to the development of extremely efficient and specific platforms for capturing CTCs, which have improved capture efficiency, purity, and sensitivity. To improve the purity and specificity of CTC capture, capture substrates like nanofiber mats need to be changed so they don’t allow nonspecific proteins to stick to them or blood cells to stick to them.

Zhili Wang et al. created an antifouling nanostructure substrate using hydrogel nanoparticles to efficiently capture circulating tumor cells (CTCs) from blood samples. The hydrogel nanoparticles were produced using a straightforward polymerization process using zwitterionic sulfobetaine methacrylate (SBMA), methacrylic acid (MAA), and N, N’-methylene bisacrylamide (MBA). SBMA can create an efficient antifouling layer on the substrate to prevent random cell adhesion. MAA can provide active carboxyl groups for attaching antibodies to enable specific capture of circulating tumor cells (CTCs). The nanostructured surface can improve the interaction between target cells and the substrate surface that has been modified by antibodies. This makes CTC capture more effective. Additionally, there was no need for any alterations to the antifouling molecules on the surface of the hydrogel nanoparticle substrate, which decreased the complexity and difficulty of preparing the substrate. The findings showed that the antibody-modified hydrogel nanoparticle substrate trapped about 87% of MCF-7 cells. Conversely, the substrate exhibited minimal adhesive capability for the nonspecific cells (K562 cells), capturing only 0.15% of the cells. 98% of the collected cells maintained high cell viability. 1–32 circulating tumor cells per milliliter were identified in the blood samples of five cancer patients, whereas no circulating tumor cells were observed in five healthy samples (**Figure 4D**) (115).

**Figure 5:**
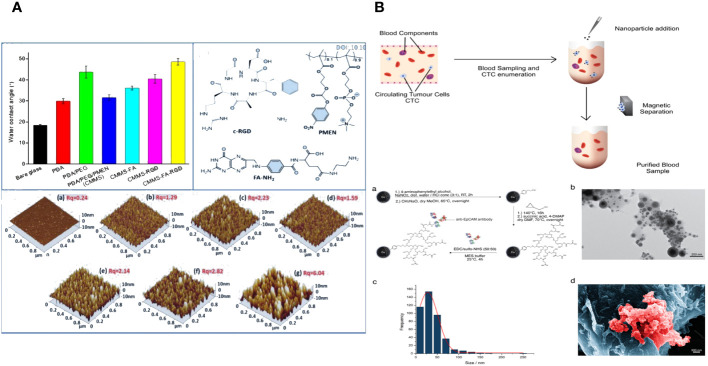
**(A)** The fabrication, blood cell repellence and tumor cell capture of ligands decorated cell membrane mimetic surface (CMMS-FA-RGD) (117). **(B)** Overview of working principle in blood from cancer patient; Nanoparticle synthesis and characterization (118).

Tong Li et al. constructed a red blood cell membrane mimetic surface (CMMS) on various substrates to prevent blood cell attachment. Tumor cell-targeting ligands, folic acid (FA) and arginine-glycine-aspartic acid (RGD) peptides, are attached to the CMMS to enhance its ability to capture tumor cells, creating the decorated surface (CMMS-FA-RGD). The CMMS consists of a self-adhesive polydopamine layer inspired by mussels, together with a non-fouling or anti-cell adhesion layer made of a phosphorylcholine zwitterion polymer and poly (ethylene glycol) (PEG) that is covalently anchored. The extended sections of the PEG chains attached to the anchored CMMS are linked with FA and RGD ligands to provide targeted binding to tumor cells. Additionally, all elements of the systematically created surfaces can be precisely controlled to enhance non-specific cell repulsion and tumor cell adhesion. The intricately designed CTC capture surface boosts the HeLa cell enrichment factor to 18000-fold by preventing over 99.999% of blood cells from adhering, leading to a high capture efficiency (91%) and capture purity (89%) from spiked whole blood samples. This method of modifying surfaces to collect tumor cells independently of the substrate and reject blood cells could offer a simple, adaptable, and cost-effective technology solution for improved cancer diagnostics and targeted therapy (**Figure 5A**) (117).

Novel magnetic nanoparticles consisting of carbon-coated cobalt (C/Co) were created and linked with anti-epithelial cell adhesion molecule (EpCAM) antibodies to investigate their antifouling and separation characteristics. The recently created C/Co nanoparticles demonstrated outstanding separation and antifouling characteristics. The tumor cells added to healthy patients’ blood samples were effectively eliminated by an interaction with an anti-EpCAM antibody. The nanoparticles showed little interaction with other blood components, such as lymphocytes or the coagulation system. On average, at least 68% of circulating tumor cells (CTCs) were eliminated from the blood samples of carcinoma patients with metastatic illnesses. The nanoparticles have the potential to stimulate the creation of a blood purification technology, like a dialysis device, to remove circulating tumor cells from the blood of cancer patients after surgery and potentially enhance their outlook (**Figure 5B**) (118).

### 3.2 Enhancement of tumor treatment

Nanoparticle-based cancer treatments have been extensively researched due to their ability to minimize side effects and enhance effectiveness (119). An ideal nanomaterial should possess prolonged blood circulation, increased accumulation in tumor tissue, and improved internalization by cancer cells (120). To accomplish this, “stealthy” coverings such as hydrophilic polymers, proteins, and cell membranes were utilized to shield nanomaterials, resulting in prolonged blood circulation and effective accumulation at tumor locations. This is achieved by a phenomenon called the increased permeability and retention (EPR) effect (121). Yet, these covert nanomaterials frequently experience diminished absorption by cancer cells, resulting in low levels within cancer cells and tissues.

One frequent technique to improve the absorption of nanomaterials by cancer cells is to incorporate targeted molecules. Adding targeting moieties like antibodies could unfortunately make nanomaterials less effective at passive targeting and extending their circulation time. It remains a significant problem to prolong the circulation of nanomaterials while simultaneously enhancing their uptake by cancer cells. A new targeting technique was created using nanoparticles designed with a responsive surface coating. This coating may be deactivated in tumor tissue under specific conditions, enabling cancer cell absorption (122). One effective approach involves attaching cancer-targeting components to nanoparticles and then applying antifouling coatings to inhibit non-specific interactions while circulating (123). Removing the shielding layer would reveal the targeted moieties and trigger cell uptake once the nanomaterials reach the tumor microenvironment.

#### 3.2.1 Enhancing tumor targeting

The most crucial variables in cancer diagnostics and therapy are tumor targetability and site-specific release. Biofouling is the unintentional accumulation of organisms, proteins, or biomolecules on the surface of metal nanocomplexes. The phenomenon is a significant concern in bioinorganic chemistry as it causes the formation of a protein corona, leading to the destabilization of a colloidal solution and triggering unwanted macrophage-driven clearance, ultimately resulting in the unsuccessful delivery of a specific therapeutic payload.

For successful tumor treatment, the nanoplatforms gathered in the tumor area must exhibit improved cellular absorption and penetration into the tumor. Antifouling nanoplatforms like zwitterionic near the tumor site may not be optimal for increasing tumor cell uptake and penetration. Furthermore, when the nanoplatforms are taken up by the tumor cells, the medication molecules they contain should be quickly released to produce their therapeutic impact. Hence, it is essential to effectively balance the antifouling characteristics and improved tumor cell uptake capability of nanoplatforms. The strategic development of smart nanoplatforms capable of reacting to the tumor microenvironment is crucial.

**Figure 6:**
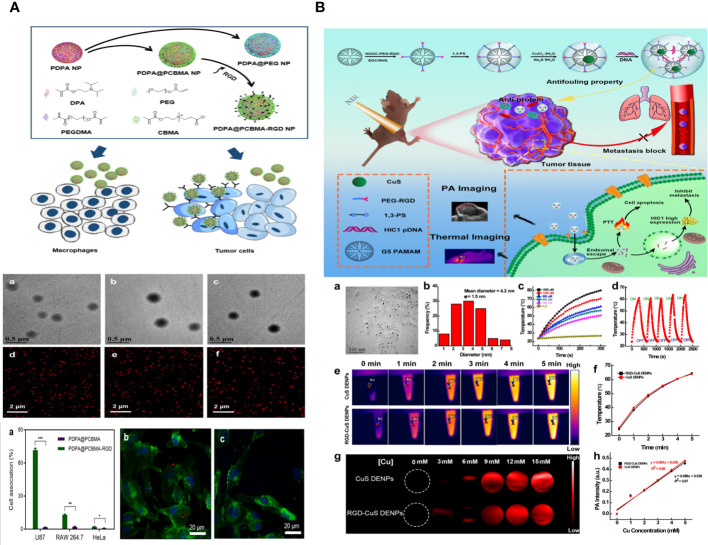
**(A)** Illustration of the preparation of PDPA@PEG, PDPA@PCBMA, and PDPA@PCBMA-RGD NPs as well as the low-fouling property and targeting ability of PDPA@PCBMA-RGD NPs. Molecular structures of the monomers [DPA, 2-(diisopropylamino)ethyl methacrylate; PEG-acrylate, poly (ethylene glycol) methyl ether acrylate; CBMA, carboxybetaine methacrylate] and the cross-linker (PEGDMA, polyethylene glycol dimethacrylate) (124). **(B)** Construction of RGD-CuS DENPs for PA Imaging and PTT/Gene Therapy of Tumors and Tumor Metastasis. After endosomal escape, the pDNAs are dissociated from the polyplexes and enter into the cell nuclei to complete the protein expression to inhibit cancer cell metastasis, while the DENPs enable the PTT of cancer cells (125).

Feng Ding et al. describe the creation of pH-sensitive and targeted polymer nanoparticles (NPs) made up of poly(2-(diisopropylamino)ethyl methacrylate) (PDPA) as the core and poly (carboxybetaine methacrylate) (PCBMA) as the outer layer. These nanoparticles are modified with cyclic peptides that include Arginine-Glycine-Aspartic Acid-D-Phenylalanine-Lysine (RGD). The polymer nanoparticles PDPA@PCBMA-RGD NPs combine the pH-responsive properties of PDPA (pKa∼6.5) with the low-fouling characteristics of PCBMA, effectively preventing non-specific interactions with RAW 264.7 and HeLa cells. PDPA@PCBMA-RGD NPs can selectively target human glioblastoma (U87) cells that express αvβ3 integrin. The pH-responsive and low-fouling characteristics of PDPA@PCBMA NPs are similar to those of PDPA@poly (ethylene glycol) (PDPA@PEG) NPs, suggesting that PCBMA can serve as a substitute for PEG in low-fouling coatings. PDPA@PCBMA NPs have carboxyl groups on their surfaces, which can be modified further, for example, by adding RGD for cell targeting. The polymer nanoparticles mentioned are novel carriers with the capability for precise therapeutic delivery (**Figure 6A**) (124).

Zhijun Ouyang and colleagues have developed functional dendrimer-entrapped CuS nanoparticles (CuS DENPs) combined with plasmid DNA encoding hypermethylation in cancer 1 (pDNA-HIC1) for the purpose of using photoacoustic (PA) imaging to simultaneously inhibit tumors and tumor metastasis. Generation 5 poly(amidoamine) dendrimers were chemically linked with 1,3-propane sultone and arginine-glycine-aspartic acid (RGD) peptide using a poly (ethylene glycol) spacer and then utilized for the controlled production of CuS nanoparticles. The RGD-CuS DENPs are well-prepared with a mean CuS core diameter of 4.2 nm, showing strong colloidal stability. They exhibit excellent absorption in the second near-infrared window, resulting in a photothermal conversion efficiency of 49.8% and exceptional PA imaging capabilities. The functional DENPs efficiently transport pDNA-HIC1 to inhibit cancer cell invasion and metastasis in the presence of serum due to their zwitterionic alteration that provides an antifouling property. The RGD-CuS DENPs/pDNA polyplexes exhibit increased anticancer effects aimed at αvβ3 integrin by combining CuS NP-mediated photothermal therapy (PTT) with pDNA delivery to suppress cancer cell metastasis. This is supported by the treatment effectiveness of a triple-negative breast cancer model in living organisms, where the suppression of both the main subcutaneous tumor and lung metastasis can be achieved (**Figure 6B**) (125).

The CD44 receptor is commonly used as a biomarker due to its overexpression in many tumors, making it a target following the discovery of the CD44 binding peptide. A. De Capua et al. incorporated the CD44 binding peptide logic into an oil core-polymer multilayer shell, considering and enhancing key aspects of drug delivery systems, including small size (as small as 100 nm), precise size distribution, drug loading capacity, antifouling properties, and biodegradability. In addition to facilitating active targeting, the oil-core-based approach allows for the transportation of both natural and manufactured medicinal chemicals. Biological experiments showed that nano capsules functionalized with CD44-binding peptide and curcumin selectively aggregate and enter cancer cells due to ligand-receptor binding, as evidenced by fluorescence tagging (126).

**Figure 7:**
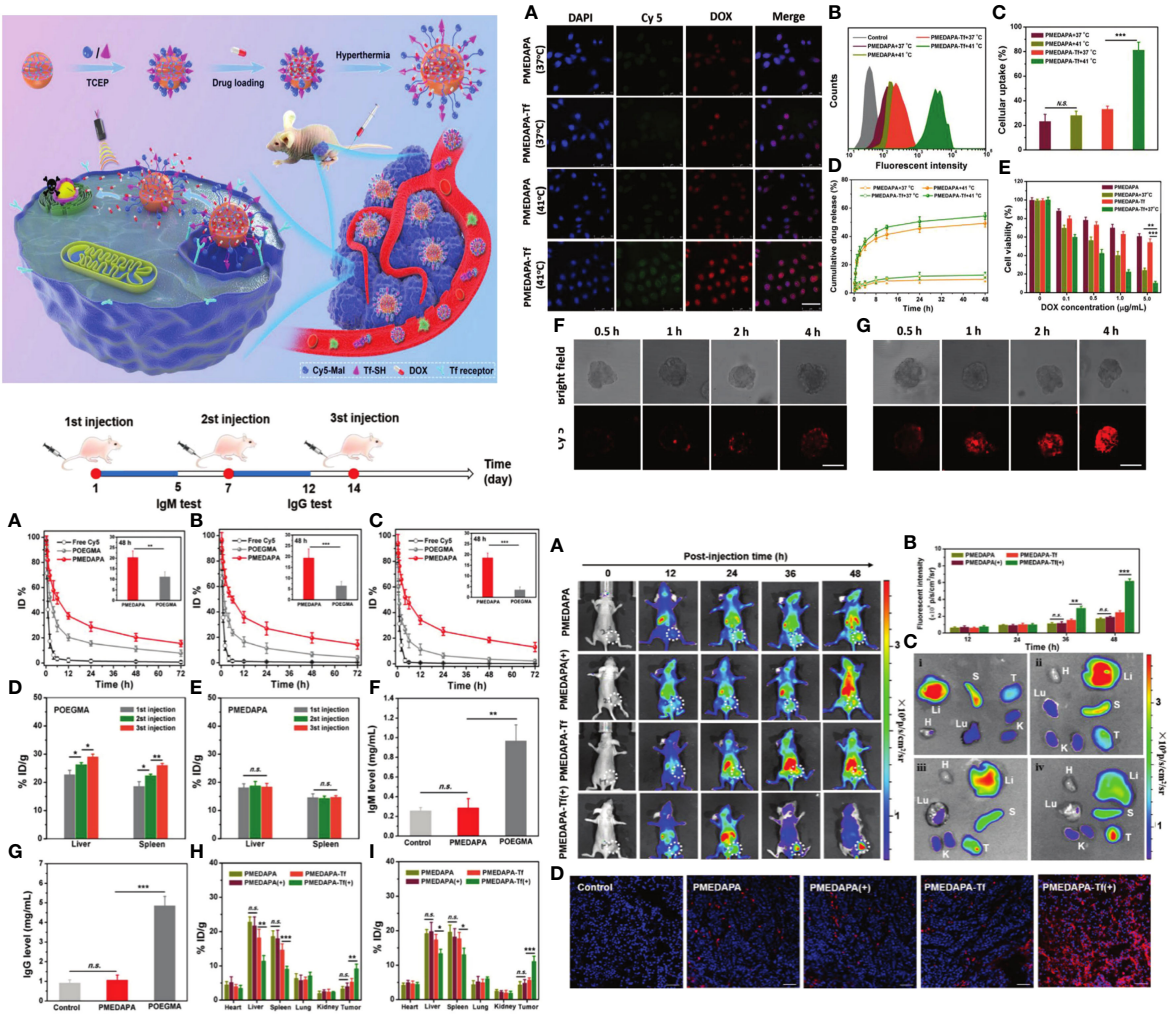
The preparation, hyperthermia-responsiveness, and antitumor therapy of doxorubicin hydrochloride (DOX)-loaded PMEDAPA-Tf nanogels (PMEDAPA-Tf@DOX). PMEDAPA-Tf@DOX shows long blood circulation without inducing the accelerated blood clearance (ABC) phenomenon. F ­urthermore, PMEDAPA-Tf@DOX exhibits enhanced tumor accumulation, penetration, and on-demand drug release with the clinically used microwave heating, leading to improved cancer therapy (127). Upper left: *In vivo* blood retention profiles of free Cy5, POEGMA, and PMEDAPA nanogels after the first injection **(A)**, second injection **(B)**, and third injection **(C)** in BALB/c mice. Upper right: Confocal laser scanning microscopy observation. Lower right: Fluorescent imaging of mice bearing HepG2 tumors. Upper left: The preparation, hyperthermia-responsiveness, and antitumor therapy of doxorubicin hydrochloride (DOX)-loaded PMEDAPA-Tf nanogels (PMEDAPA-Tf@DOX).

A sulfamide-based zwitterionic monomer was created and utilized to produce a range of polysulfamide-based nanogels known as PMEDAPA, which serve as drug carriers for efficient cancer treatment. PMEDAPA nanogels have been shown to have extended blood circulation time without causing the accelerated blood clearance effect. PMEDAPA nanogels can sensitively react to hyperthermia by modifying the degree of crosslinking. Upon modification with transferrin (Tf), the nanogels (PMEDAPA-Tf) demonstrate protected tumor targeting at normal body temperature and restored tumor targeting at elevated body temperature, resulting in increased tumor formation. PMEDAPA-Tf nanogels exhibit enhanced penetration in 3D tumor spheroids and quicker drug release under hyperthermia conditions compared to normothermia. When mild microwave heating (≈41°C) is applied, the drug-loaded PMEDAPA-Tf nanogels demonstrate significant tumor inhibition in a humanized orthotropic liver cancer model. The work introduces a new hyperthermia-responsive zwitterionic nanogel that can enhance tumor accumulation and trigger medication release using microwave heating for cancer treatment (**Figure 7**) (127).

#### 3.2.2 Enhancing drug delivery to tumors

Malignant tumors are a leading cause of death globally, and chemotherapy is a widely used method in clinical therapies (128). Nevertheless, the vague delivery and limited absorption of anticancer medicines in their free form typically result in severe adverse effects on healthy tissue and reduced therapeutic effectiveness (129). Nanoscale drug delivery systems (DDSs) have been created to improve the bioavailability of anticancer medications. They promote the transport of therapeutic cargos to tumor sites, which increases the drugs’ effectiveness and reduces systemic toxicity. Common examples of drug delivery systems include liposomes, micelles, polymers, dendrimers, and organic or inorganic nanoparticles (130). Drug delivery systems have demonstrated significant potential to enhance the solubility and stability of the medicinal substances they carry. During circulation, cells and plasma proteins in the blood can adhere to the drug delivery systems (DDSs) and cause them to be removed by the mononuclear phagocyte system (MPS) (131). The primary focus for developing multifunctional nanocarrier systems should be on preventing environmental biomolecules from adhering nonspecifically to nanocarriers. This will help decrease the accumulation of anti-cancer drugs in healthy metabolic organs and enhance drug bioavailability. Various non-fouling materials and coatings, including polysaccharides, ethylene glycol-based polymers, and oligomers, which are typically well hydrated and carry neutral or weakly negative charges, have been used to accomplish this objective (132). Zwitterions, known for their hydrophilic nature and low interfacial energy, have been shown to be excellent options for preventing fouling in drug delivery systems (111). A significant obstacle to effective medication administration and killing tumor cells is the need for efficient uptake by tumor cells and escape from endosomes after the nanocarriers accumulate at tumor sites (133). Evidence shows that changing the surface charge of nanocarriers from negative to slightly positive after circulating in the blood can enhance cellular uptake through electrostatic interaction-mediated internalization. Additionally, the “Proton Sponge” effect in acidic cellular compartments like endo-lysosomes (pH 4.0–5.5) can promote the successful escape of therapeutic substances from endosomes before reaching full effectiveness. Functional components in an intelligent drug delivery system must be capable of achieving anticipated changes in specific tumor settings to fulfil all criteria at the same time.

Poly(2-oxazoline) s are a new type of polymer that is becoming more important in the field of biomedical sciences. Currently, the majority of research on poly(2-oxazoline)-drug conjugates has been centered on poly(2-ethyl-2-oxazoline) (PEtOx), which is a biocompatible water-soluble polymer that shares biological characteristics with polyethylene glycol. The hydrophilic poly(2-methyl-2-oxazoline) (PMeOx) has superior anti-fouling capabilities compared to PEtOx, suggesting a higher potential for developing polymer therapies. Ondrej Sedlacek and colleagues developed a drug delivery method using a linear PMeOx with a large molar mass of 40 kDa to take advantage of passive accumulation in tumors through the improved penetration and retention effect. Doxorubicin, an anti-cancer medication, is linked to a polymer carrier through an acid-sensitive hydrazone bond, enabling its release in response to the tumor’s pH. The *in vitro* investigation shows that the PMeOx-doxorubicin combination is effectively taken up by cells by clathrin-mediated endocytosis, has pH-sensitive drug release, and exhibits strong toxicity against B16 melanoma cells. The properties of the PMeOx carrier were compared to those of PEtOx systems. PMeOx showed higher drug loading, better cellular uptake, improved anti-fouling properties, and enhanced *in vitro* anti-cancer efficiency compared to PEtOx. The study shows that PMeOx has the potential to be a versatile platform for creating new medication delivery systems (134).

Yu-Lun Li and colleagues created a nanofibrous delivery system that is activated by near-infrared (NIR) light. The system is made of zwitterionic poly (2-methacryloyloxyethyl phosphorylcholine)-b-poly(ϵ-caprolactone) (PMPC-b-PCL) and contains indocyanine green (ICG) and doxorubicin (DOX) for combined photothermal therapy and chemotherapy. The nanofibrous mat displays hydrophilic properties and effective antifouling performance. ICG can convert near-infrared light into thermal energy, raising the temperature above 45°C under gentle near-infrared irradiation. The thermal energy speeds up the release of DOX from the nanofibrous mat by softening the nanofibers, suggesting that drug release can be regulated, activated, or deactivated via light stimulation. Furthermore, this light-induced thermal energy and release activity help increase cell death. Confirmation of increased drug release with light exposure is supported by intracellular DOX distribution. The results show that the produced light-triggered drug release nanofibers, known as LDDS, are biocompatible, antifouling, and exhibit superior combinational chemotherapy and photothermal therapy (135).

**Figure 8:**
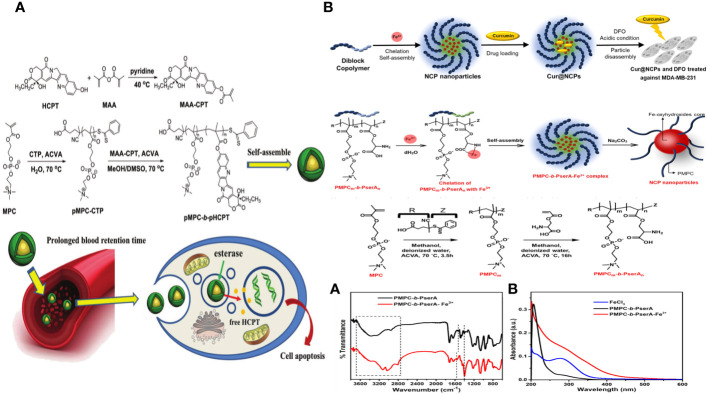
**(A)** Schematic diagram of polyprodrug amphiphiles pMPC-b-pHCPT from synthesis, self-assembly, to delivery *in vivo* (136). **(B)** Schematic of the Synthesis of Curcumin-Loaded NCPs (Cur@NCPs) and Their Disassembly and Release of Curcumin, which Induces the Apoptosis of Breast Cancer Cells (MDA-MB-231). The diblock copolymer PMPC-b-PserA self-assembles into NCP nanoparticles via complexation of Fe3+ and PserA. Cur@NCPs are produced by partitioning of curcumin into the hydrophobic particle core via an oil-in-water emulsion. Addition of the chelating agent DFO in an acidic solution causes disassembly of the Cur@NCPs, curcumin release, and cell apoptosis (137).

A polymeric drug delivery system called “polyprodrug amphiphile” (pMPC-b-pHCPT) was created for long-lasting cancer treatment. The copolymer is synthesized using a two-step reversible addition-fragmentation chain transfer polymerization of the zwitterionic monomer MPC and an esterase-responsive polymerizable prodrug, methacrylic anhydride-CPT. This diblock polymer consists of antifouling (pMPC) and bioactive (pHCPT) segments, with the medication serving as a foundational component for constructing the polymer structure. The polymer can spontaneously form micelles of varying sizes by adjusting the ratio of MPC/HCPT due to its unique amphiphilic properties, all under physiological conditions. The outer pMPC shell is highly hydrophilic to create a dense hydrate layer that prevents nonspecific protein adsorption, a major factor in the quick removal of nanoparticles in the body. This helps drugs accumulate in tumor sites by enhancing permeability and retention. Multiple measurements validate the structure of the polyprodrug amphiphile. The study also examines the micelles’ ability to resist albumin adsorption, extend plasma retention time, accumulate in tumor areas, and exhibit anticancer properties through *in vitro* and *in vivo* experiments. This amphiphile compound shows promise as an effective agent for long-lasting cancer treatment through passive targeting (**Figure 8A**) (136).

Pin-Chun Chen and colleagues created a new nanoscale coordination polymer by utilizing the diblock copolymer poly (2-methacryloyloxyethyl phosphorylcholine)-block-poly (serinyl acrylate) (PMPC-b-PserA). They showcased its ability to encapsulate a hydrophobic drug and release it upon activation, leading to programmed cell death in breast cancer cells in a laboratory setting. The zwitterionic PMPC block was influenced by the antifouling properties of cell membranes, whereas the PserA block was inspired by the amphoteric nature of amino acids found in proteins. The polymer was created using reversible addition-fragmentation chain transfer polymerization. A combination of the polymer and FeCl3 formed nanoparticles through the interaction of Fe3+ with PserA and the hydrophilic PMPC block located on the particle’s surface. When the molar ratio of Fe3+ to serA was 3:1, the particles had a hydrodynamic diameter of 22.2 nm. Curcumin, a hydrophobic polyphenol, was encapsulated in particles as an oil-in-water emulsion. The encapsulation effectiveness was 99.6%, and the particle loading capacity was 32%. Curcumin release was activated by the addition of deferoxamine, an FDA-approved drug that binds to Fe3+; the efficiency of curcumin release improved with larger deferoxamine concentrations and lower pH levels. Curcumin release was activated, leading to apoptosis in human triple-negative breast cancer cells. Cell viability dropped to 34.3% following 24 hours of treatment with curcumin-loaded nanoparticles and deferoxamine, compared to over 80% viability without deferoxamine to initiate drug release. This nanoscale coordination polymer is ideal for delivering anticancer drugs due to its biocompatibility, adjustable composition and size, high hydrophobic drug loading capacity, and ability to release drugs in response to triggers (**Figure 8B**) (137).

#### 3.2.3 Enhancing the efficacy of radiotherapy for tumors

Radiation therapy is a commonly used non-surgical treatment for cancer patients in clinics because of its deep tissue penetration (138). High-energy radiation, often X-rays, can be used to target and destroy DNA double strands in cancer cells while minimizing damage to adjacent healthy tissues (139). Hence, a viable approach is to introduce radiosensitizer molecules that can interact with X-rays into certain cells or tissues to target the radiation dose (140). Nanoparticles offer an advantage over traditional molecular radiosensitizers in that they may be engineered to accumulate preferentially in tumor tissues by leveraging the increased permeability and retention (EPR) effect, the advent of nanotechnology has facilitated the aforementioned developments (141). Various high-atomic-number metal-based nanomaterials such as gold (142), bismuth (143), gadolinium (144), and hafnium (145) have been thoroughly researched for their potential as radiosensitizers. Gold nanoparticles (Au NPs) are extensively researched for their inherent radiosensitive properties stemming from their high atomic number and superior biocompatibility when compared to other high atomic number materials (146). Au NPs have the added benefit of serving as contrast agents for guided therapy and can be effectively combined with nanocarriers for other medicines (147). While the radiosensitivity of gold nanoparticles is well recognized, difficulty still exists for their therapeutic application. An ideal radiosensitizer should have quick elimination from the body to reduce long-term toxicity and high uptake in tumor tissue and cells, including the cell nucleus, to reach a concentration useful for therapy (148).

**Figure 9:**
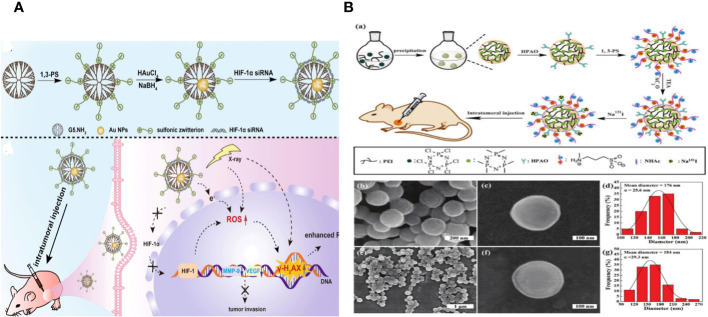
**(A)** (a) Schematic illustration of the preparation of {(Au0) 25-G5.NH2-PS20}/siRNA polyplexes. (b) The mechanism of {(Au0) 25-G5.NH2-PS20}/siRNA polyplexes for combined endogenous and exogenous sensitization of tumor RT via HIF-1α gene knockdown and Au NPs, respectively (149). **(B)** (a) Schematic illustration of the preparation of PNS.NHAc-HPAO (131I)-PS. SEM images of (b, c) PNSs and (e, f) PNS.NHAc-HPAO-PS spheres. (d, g) show the size distribution histograms of PNSs and PNS.NHAc-HPAO-PS, respectively (150).

Chao Yang and colleagues wanted to create an advanced nano system to improve radiation therapy for tumors by utilizing both internal and external sensitization methods using Au DENPs. Amine-terminated G5 dendrimers were modified using 1,3-propanesultone (1,3-PS) to make them antifouling and then encapsulated with Au NPs in this design. We thoroughly analyzed the Au DENP vector system regarding its structure, shape, content, stability, cytotoxicity, gene compaction and silencing capacity, and *in vitro* dual sensitization-induced anticancer efficiency. The nano system was used for radiation therapy on a xenografted tumor model to investigate the dual sensitization effect. This work is the first to combine endogenous and exogenous sensitization using dendrimer nanotechnology to enhance tumor radiation therapy (**Figure 9A**) (149).

The study describes the creation of versatile poly(cyclotriphosphazene-co-polyethylenimine) nanospheres (PNSs) tagged with radioactive 131I for tumor treatment guided by single photon emission computed tomography (SPECT) imaging. Wei Zhu et al. prepared PNSs by crosslinking branched polyethylenimine (PEI) with hexachlorocyclotriphosphazene through a nucleophilic substitution reaction. The particles were then modified with 3-(4′-hydroxyphenyl) propionic acid-OSu (HPAO) for 131I labelling, treated with 1,3-propane sulfonate (1,3-PS) to provide antifouling properties, and finally acetylated to label the remaining surface amines with 131I. The obtained PNS.NHAc-HPAO (131I)-PS particles are thoroughly characterized. The multifunctional PNSs, averaging 184 ± 29.3 nm in size, have strong antifouling capabilities, efficient 131I labelling (76.05 ± 3.75%), and exceptional radio stability and colloidal stability. The PNS. NHAc-HPAO (131I)-PS spheres have features that allow for more effective SPECT imaging and treatment of a xenografted tumor model *in vivo* compared to the PEI. NHAc-HPAO (131I)-PS material (**Figure 9B**) (150).

#### 3.2.4 Enhancing PTT treatment of tumor

Significant advancements have been achieved in the research of novel cancer treatment techniques, particularly in the areas of photothermal therapy (PTT) (151) and gene therapy (152). PTT uses chemicals that convert light into heat within the near-infrared (NIR) range to induce hypothermia in cancer cells (153). PTT irreversibly damages cancer cells by disrupting the cell membrane structure and breaking down DNA, RNA, and proteins in the nucleus (154). PTT agents can be used for photoacoustic imaging when exposed to laser irradiation (155). PTT of cancer in the second NIR window (NIR-II, 1000–1700 nm) has greater potential compared to PTT in the first NIR window (650–950 nm) because of lower tissue absorption and scattering, enhanced tissue penetration, and maximized allowable exposure (156).

**Figure 10:**
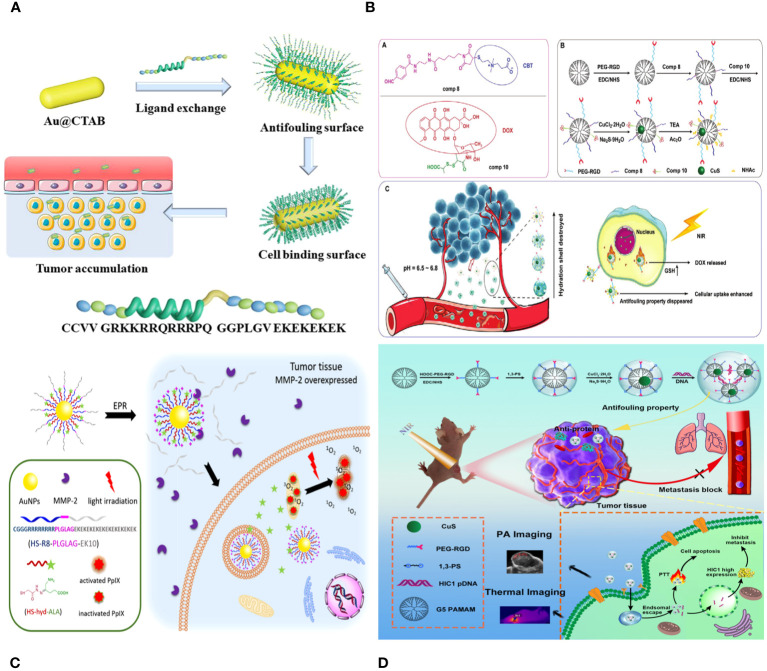
**(A)** Schematic illustration to show the preparation of multifunctional peptides capped AuNRs and their tumor accumulation *in vivo* for photothermal therapy (157). **(B)** Schematic illustration of the synthesis of A) benzaldehyde–thiolated CBT and DOX-DTPA conjugate, B) functionalized CuS DENPs, and C) their therapeutic applications *in vivo* (158). **(C)** Schematic Illustration of Enzyme and pH Dual-Sensitive ALA-Conjugated AuNPs for Targeted PDT (159). **(D)** Construction of RGD-CuS DENPs for PA Imaging and PTT/Gene Therapy of Tumors and Tumor Metastasis. After endosomal escape, the pDNAs are dissociated from the polyplexes and enter into the cell nuclei to complete the protein expression to inhibit cancer cell metastasis, while the DENPs enable the PTT of cancer cells (125).

Liming Wu et al. have tackled the issue by creating an enzyme-sensitive zwitterionic stealth peptide coating that can react to matrix metalloproteinase-9 (MMP-9), a protein that is highly produced in the milieu around tumors. The peptide has a Tat sequence that can get into cells, an MMP-9 sequence that can be cut, and a zwitterionic antifouling sequence. Applying this coating to shield photothermal gold nanorods (AuNRs) led to responsive AuNRs with improved systemic circulation lifetime and increased cellular absorption in tumors. This enhancement resulted in a noticeable improvement in photothermal therapeutic effectiveness in mouse models. The findings indicate that multifunctional peptide-coated gold nanorods (AuNRs) responsive to MMP-9 show potential as nanomaterials with prolonged systemic circulation and increased accumulation in tumor tissue, offering a more targeted and effective approach to tumor therapy (**Figure 10A**) (157).

Creating stimulus-responsive nanomedicine with improved tumor targeting for combination therapy is still a significant issue. A novel antifouling-dendrimer-based nanoplatform with both pH- and redox-responsive properties is presented to address this issue. Generation 5 (G5) poly(amidoamine) dendrimers are altered by attaching a targeting ligand cyclic arginine-glycine-aspartic acid (RGD) peptide using a polyethylene glycol (PEG) spacer and a zwitterion of thiolated N, N-dimethyl-cysteamine-carboxybetaine (CBT) through a pH-responsive benzoicimine bond to create G5. NH2-PEG-RGD-CBT conjugates. Doxorubicin is connected to the functional G5 dendrimers using a redox-responsive disulfide connection, and then CuS nanoparticles are trapped inside the dendrimers. The functional dendrimer-CuS nanohybrids, with a CuS core size of 3.6 nm, exhibit strong antifouling characteristics and exceptional photothermal conversion efficiency in the second near-infrared range. The nanohybrids can change from a neutral surface charge to a positive one in the tumor area in a slightly acidic environment by breaking the benzoicimine bond, enhancing their uptake by cells. Additionally, the redox-sensitive disulfide bond allows for the quick release of DOX within tumor cells for its therapeutic impact. The dendrimer-CuS nanohybrids, in conjunction with the CuS cores, provide improved combined chemotherapy and photothermal therapy for tumors (**Figure 10B**) (158).

#### 3.2.5 Enhancing PDT treatment of tumor

Photodynamic therapy (PDT) is gaining interest as a possible method for treating tumors and other disorders without the need for intrusive procedures (160). During photodynamic therapy (PDT), the photosensitizer (PS) injected can be activated by particular light to generate reactive oxygen species (ROS), which are highly harmful to tumor cells (161). Due to its noninvasive nature and few side effects, PDT is gaining increasing attention for treating tumors.

5-Aminolevulinic acid (ALA) is an approved medicinal drug by the U.S. FDA that serves as the precursor of the photosensitizer protoporphyrin IX (PpIX). Efficiently delivering ALA remains a significant issue due to its hydrophilic nature, which hinders its recognition and selective accumulation in tumor cells. This work developed nanocarriers that are sensitive to both matrix metalloproteinase-2 (MMP-2) and pH and designed for the specific delivery of ALA. The nanocarriers were created by modifying gold nanoparticles (AuNPs) with hydrazone-linked ALA and MMP-2-activatable cell-penetrating peptides (CPPs). The cationic cell-penetrating peptide RRRRRRRR (R8) was protected by the zwitterionic stealth peptide EKEKEKEKEKEKEKEKEKEKEK (EK10) using the MMP-2 substrate peptide PLGLAG. The zwitterionic stealth peptide EK10 was created to provide ALA prodrug nanocarriers with potent antifouling properties and extended circulation duration. When the protected cationic CPP R8 reaches the tumor tissue, it can be triggered by MMP-2 overexpression in the tumor microenvironment, leading to increased cellular absorption of ALA. The study showed that tumor-microenvironment-sensitive ALA prodrug nanocarriers have the potential to be used in targeted photodynamic cancer therapy based on their drug loading and release, cellular uptake, PpIX generation and accumulation, photodynamic cytotoxicity, and photodynamic tumor inhibition results (**Figure 10C**) (159).

#### 3.2.6 Enhancing gene delivery to tumors

Gene therapy has emerged as a leading approach for treating both genomic and non-genomic disorders, as ongoing investigations consistently show (162). Currently, numerous therapeutic genes are undergoing clinical trials, with some already receiving approval for commercialization (163). Safe and effective carriers are essential for delivering therapeutic genetic molecules to specific places and ensuring proper transcription and translation into functional proteins (164). Current gene delivery vectors consist of viral vectors, non-viral cationic polymers, and inorganic metal compounds (165). Viral vectors have demonstrated improved gene delivery efficiency, but their immunogenicity prevents repeated administration, hindering their clinical applicability. Insertional mutation hazards and challenges in mass production further restrict their use. Cationic polymers can condense negatively charged gene molecules and aid in their cellular absorption, while positively charged macromolecules exhibit significant cytotoxicity and tend to interact with other biomolecules in the blood (166).

Dendrimers are a diverse category of man-made large molecules made up of central cores, branching units, and outside functional groups with a clearly defined molecular structure and makeup. Dendrimers have interior cavities that can be filled with metal or other inorganic nanoparticles or anticancer medicines, and their outer surface can be altered with different functional groups or moieties for diverse nanomedicine uses. Dendrimers can encapsulate Au NPs inside them and can be partially functionalized with poly (ethylene glycol) (PEG), β-cyclodextrin, or zwitterions on their surfaces for improved gene delivery purposes. Zwitterion-decorated dendrimer-entrapped gold nanoparticles can efficiently transport genes in the presence of serum, thanks to their outstanding antifouling characteristics. In addition, dendrimer nanotechnology was utilized to create dendrimer-assembled MoS2 nanoflakes and Au nano stars for delivering siRNA to achieve combined photothermal therapy and gene therapy for tumors (**Figure 10D**) (125).

Zwitterionic materials have been involved in therapeutic gene delivery. Dai et al. proposed synthesizing a double thermoresponsive SB-based ‘‘ABA” structured zwitterionic triblock polymer via ATRP. In this structure, ‘‘A” represents MPDSAH and ‘‘B” represents PEG analogue MEO2MA with LCST behavior. This approach aimed to prevent the charge neutralization of cationic polymers from causing the precipitation of complexes after condensing negatively charged nucleotide molecules. The SB-based MPDSAH condensed DNA molecules by using their quaternary ammonium cations, while their sulfonic anions remained unreacted to prevent inter-complex coacervation. The spatial barrier between the positive and negative charges of MPDSAH was decreased by the presence of three methylene groups, allowing for efficient complexation of DNA with PMPDSAH. However, the presence of DNA molecules caused disruption to the ion pairs in MPDSAH, leading to the elimination of their UCST. The LCST remained intact with a minor increase (167).

### 3.3 Application of antifouling materials in medical devices

Protein and bacteria biocontamination significantly affect biosensors, contact lenses, medical transplantation equipment, and maritime ships. Hence, preventing biofouling and creating innovative antifouling materials are crucial in practical applications. PEG and its derivatives are currently the most commonly used compounds for combating biological contamination (168). PEG materials are prone to oxidation in biological settings and exhibit low stability. Zwitterionic polymers are now a perfect alternative to PEG. Zwitterionic polymer materials’ mechanism for combating biological pollution has been extensively researched and acknowledged. Zwitterionic polymers possess significant dipole moments and numerous hydrophilic groups, including carboxyl, sulfonic acid, and amine. Many water molecules can surround these groups, creating a strongly bonded hydration layer on the material’s surface. This can result in the material becoming super hydrophilic and effectively preventing the adsorption of proteins and other biomolecules on its surface (169). Zwitterionic polymers have been utilized in creating different biomedical materials, including polyurethanes, poly (vinylidene fluoride), and metal compounds. This imparts outstanding antifouling qualities to the biological substrates. Zwitterionic polymers are now considered the flagship of a novel class of antifouling materials.

Cai et al. created a PCB-based hydrogel covering to enhance the blood compatibility of activated carbon (PAC). Antifouling ability is crucial for the advancement of medical metal implants. Zwitterionic block copolymers can be attached to various metal surfaces to provide effective antifouling characteristics. This effective antifouling technique is commonly employed in biomedical metal implants and equipment (170).

Takai et al. created a range of poly (MPC-b-(MPTSSi-r-MPTMSi) block copolymers and effectively used them to coat glass substrates with consistent polymer layers. Zwitterionic block copolymers have high protein tolerance and broad pH stability ranging from pH 2 to 9. There is no need for pre-treatment of the hydrophilic surface that the zwitterionic block copolymer coating creates. This makes it acceptable for use in medical and environmental purification equipment such as biosensors, sanitary ware, and water purification systems (171).

Mi et al. created a thermosensitive, multifunctional ABA triblock copolymer hydrogel for wound treatment. Block B has a positively charged and hydrolyzable betaine ester, together with antibacterial medicines. The copolymer solution was administered to the wound and quickly transformed into a gel upon reaching body temperature. Antibacterial medications can lower the likelihood of wound infection, while their positive charge can enhance cell adhesion in tissue regeneration (172).

## 4 Challenges and prospects of antifouling materials in oncology applications

This review discusses many antifouling materials and their applications in medication delivery, anticancer therapy, biomedical diagnosis, and antifouling coatings. Antifouling material drug carriers excel in drug delivery with their high drug loadings, encapsulation efficiency, low clearance rates, and extended circulation times. The smaller size and higher stability of antifouling materials enhance anticancer efficacy by promoting quick internalization of cells, leading to increased cell uptake and improved antitumor effects. Imaging, carbon dots, and magnetic materials can be used as antifouling materials for early disease diagnosis and therapy in the field of biomedical diagnosis. The nanoscale size and high graft density of antifouling coatings provide exceptional stability and lubricity, which enhance the advancement of antifouling coatings and wear-resistant materials. In conclusion, antifouling materials have had a significant impact on biomedical research.

Despite significant advancements in antifouling materials for biomedical purposes, there are still significant hurdles that need to be overcome in the future. Only a small number of these techniques have been successful in clinical settings, suggesting that there are still obstacles, such as intricate synthesis procedures and uncertain drug delivery systems. Secondly, in the context of drug distribution, the sudden disintegration of drug carriers might lead to a rapid and uncontrolled release of medicines. To address this issue, it is crucial to develop a controlled-release medication system that incorporates numerous binding forces. Improving precise, targeted regulation for tumor treatment while minimizing harm to healthy tissues is a significant challenge. Enhancing the sensitivity and accuracy of imaging diagnostics for biomedical purposes is an ongoing area of research. Antifouling compounds have advanced significantly in their use for preventing fouling. Nevertheless, their anti-fouling capabilities have a restricted duration, and their industrial advancement has not been put into practice. Therefore, it is crucial to provide durable antifouling coatings for use in intricate environments.

The impedance of the modified electrode surface will increase due to the low electrical conductivity of PEG, resulting in a decrease in detection sensitivity. Because of the limits that these anti-fouling materials have by their very nature, it is very important to carefully study the nature of non-specific adsorption. When designing the electrode substrate, it’s important to think about how other modified materials and anti-fouling elements will be charged all over the electrode base, since electrostatic adsorption isn’t selective or directional.

The utilization of cell membrane coating technology is highly advantageous for researchers due to its integration of both natural and synthetic components. The development of these platforms for the isolation of CTCs was intricate and consumed a significant amount of time. This involved modifying antifouling coatings, crosslinking multiple chemical agents, and conjugating targeted molecules. These processes can result in variations between batches and potentially impact the accuracy of detection. The majority of cell-based nanoparticles designed to target tumors are currently in the clinical phase, mostly due to their successful attainment of biocompatibility. Model medicines such as doxorubicin, cisplatin, and paclitaxel are widely recognized for their therapeutic effectiveness. However, it is important to note that these drugs have challenges in terms of their pharmacokinetics, biodistribution, and the significant issue of drug resistance. Natural targeting enhances medication accumulation at the intended location, allowing it to remain in the bloodstream for an extended duration without being detected by mononuclear phagocytes. The pre-existing membrane proteins on the cell membrane-coated nanoparticles offer these benefits with minimal labor-intensive production compared to the conditions needed for immunonanoparticles. Every cell has a certain advantage that gives a CMCNP an added benefit, but they also have certain limits. RBCs have prolonged circulation, allowing them to evade RES, although they are ineffective at actively targeting them. White blood cells (WBCs) possess the capacity for homologous targeting, enabling them to selectively target specific tumors. Platelets, on the other hand, exhibit the ability to survey tumor sites and detect damage, thereby stimulating the immune system. On the other hand, cancer cells possess homologous adhesive ability or active tumor targeting, albeit with a shorter circulation time in the bloodstream. Stem cells possess tumor-targeting capability, albeit with limited specificity. Additionally, bacterial membranes possess immune-evoking properties. However, the process of extracting the membrane from these cells remains laborious due to the need for peptidoglycan removal. Based on the requirement, the combination of two membranes can be efficiently achieved to create hybrid cells that can possess the therapeutic advantages of both cells.

Novel antifouling materials could be enhanced to respond to specific stimuli in the tumor microenvironment, such as pH, redox status, or enzymes. This would help regulate the aggregation and surface charge of nanomaterials, leading to better tumor diagnosis. To enhance CTC capture, magnetic colloidal materials can be customised with antifouling materials in addition to the nanofiber system to enhance the purity, efficiency, and integrity of captured cancer cells for further biochemical investigation. Nanomaterials can be modified with antifouling materials to integrate therapeutic drugs or act as active agents under specific conditions, enhancing the theranostics of cancer cells by combining imaging functionalities. The future direction of electrochemical biosensors is focused on quickly detecting substances within a living organism. Therefore, it is crucial to create a durable anti-fouling material that possesses hydrophilicity, electroneutrality, and robust conductivity all at once. Zwitterionic inorganic or organic polymers can be designed to have certain hydrophilic, electroneutral, and strong resistance to enzymatic breakdown. Zwitterionic polymers can enhance conductivity by including conjugated groups or copolymerizing with conductive polymers. Cell membranes offer numerous therapeutic benefits due to their biocompatibility. The method eliminates the need for organic solvents, ligands, chemical conjugative moieties, and highly reactive chemical agents to create a specific nanocomposite. It is environmentally benign, exhibits a visible emission spectrum, and possesses outstanding fluorescence qualities suitable for many studies. Despite significant progress with cell membrane-coated nanoparticles, there are still difficulties that need to be addressed. The field is immature and has not yet reached the translational stage, transitioning from laboratory research to clinical trials. It requires efficient and easily reproducible techniques that can be scaled up. Additional *in vivo* assays of the modified nanomaterials are necessary for preclinical applications, including investigations into their biodistribution, metabolic pathways, and long-term biosafety profiles. Enhancements can be achieved by thoroughly grasping the tumor microenvironment, tumor metabolism, and tumor metastasis to better create adaptable nanoplatforms for improved diagnostic purposes.

The utilization of self-assembled supramolecular structures, such as surfactant/polymer micelles, (micro)emulsions, vesicles/liposomes, and layer-by-layer assemblies, in the rational design of drug delivery systems has garnered significant attention in recent years as a means to enhance treatments (173). Self-assembled supramolecular structures play a crucial role in drug delivery by enhancing the physicochemical, pharmacokinetic, and pharmacodynamic properties of drugs, thereby facilitating efficient delivery to specific locations in the body (174). The process of supramolecular production of (poly)zwitterions, such as micellization, vesiculation, and polymersome formation, is primarily influenced by electrostatic interactions. Nevertheless, it is important to consider the entropy loss of polymer chains that occurs during this process (175). The compensation between enthalpy and entropy is of significant importance in the self-assembly mechanism of polyzwitterions. The enthalpy term mostly benefits the process due to the interactions between water molecules. The interactions between water molecules, which arise from the cohesive force, are enhanced by the removal of (poly)zwitterions from the aqueous phase, resulting in a reduction of weaker interactions between water molecules and hydrophobic species. The electrostatic interactions between the positively and negatively charged pieces of the poly-zwitterion are identified as another advantageous factor in the enthalpy. The primary entropy factor that hinders the process is the arrangement of (poly)zwitterion molecules in the bilayer phase, which restricts their mobility and thus leads to a decrease in entropy (176).

Functionalized anti-fouling peptides have gained significant popularity as anti-fouling materials due to their inherent biocompatibility. Consequently, they have found extensive application in electrochemical assays for tumor markers. The anti-fouling activity of peptides can be attributed to their strong hydration, which is facilitated by the presence of polar functional groups and the high hydrogen bonding ability of zwitterionic charges. Hydrophilic and amphiphilic peptides, without charge, exhibit anti-fouling characteristics. For example, peptides incorporating the EK motif were engineered to possess electroneutrality in their anti-fouling region (57). It is possible to construct anti-fouling peptides that are either electrically neutral or hydrophilic in nature. The aforementioned benefits render peptides a highly suitable option for the development of biodegradable anti-fouling products (58, 177).

Although innovative materials show promise in biological medicine, research on their structures and characteristics is still in the early stages, indicating that there is a significant distance to go before clinical application can be achieved. A critical issue is streamlining the synthesis process and cutting down on the expenses associated with mass production. Additional research is required to address this significant obstacle in the upcoming period. The rising need for accurate drug delivery has led to the use of multiple stimuli-responsive drug delivery systems (DDSs). These systems can transport drugs to specific locations without premature leakage and release them precisely, even at targeted cell organelles, to minimize side effects and maximize therapeutic benefits. Currently, there are fewer antifouling materials for gene vectors compared to medication carriers. This indicates a significant potential for the advancement of flexible antifouling materials used in delivering therapeutic genes. Considering this viewpoint, it is important to focus on improving the transfection efficiency of therapeutic genes using antifouling materials in delivery systems. Overall, further research is required to enhance the performance of antifouling materials in various aspects such as biocompatibility, biodegradability, drug loading capacity, low cytotoxicity, environmental responsiveness, specific targeting ability, and other specific requirements for clinical use.

## 5 Conclusion

This article provides a summary of the design and implementation of antifouling changes on materials used for specific cancer diagnostics and treatment, such as polymers, peptides, proteins, and cell membranes, among others. The antifouling modifications enhance the materials’ ability to resist nonspecific protein adsorption and cell adhesion, thus protecting the targeting ability by preventing the formation of protein corona. This leads to highly targeted tumor diagnosis and therapy by reducing background cell binding. Moreover, multifunctional platforms like dual antifouling platforms or coexistent platforms combining antifouling and enhanced targeting capacity might improve the efficiency and purity of targeted tumor detection and therapy. These findings showed that antifouling changes could be crucial for the precise and efficient targeting of tumor detection and therapy. New fields often face challenges in efficiently combining raw materials, similar to the difficulties encountered in antifouling material coating methods. Despite ambiguity in material origins and stability concerns between synthesized particles and natural entities, the significant medicinal potential they offer is hindered. The increasing number of patents and new ideas in the sector suggests that this innovative biomimetic method has the potential to become the next generation of treatments.

## Author contributions

YZ: Writing – original draft, Writing – review & editing. CS: Supervision, Writing – review & editing.
